# Wall Deformation and Minimum Thickness Analysis in Micro-Milled PMMA Microfluidic Devices: A Comparative Study of Milling Strategies

**DOI:** 10.3390/mi16121308

**Published:** 2025-11-21

**Authors:** Ferah Sucularlı, Ülke Şimşek

**Affiliations:** 1 ASELSAN Inc., Radar Electronic Warfare Systems (REHİS), Ankara 06370, Türkiye; fsucularli@aselsan.com.tr; 2 Roketsan Missiles Industries Inc., Ankara 06780, Türkiye

**Keywords:** micro-milling, PMMA, milling strategy, wall deviation angle, average wall thickness, minimum wall thickness

## Abstract

Polymethyl methacrylate (PMMA) is widely used in microfluidic device fabrication due to its chemical resistance, low cost, optical transparency, and manufacturing compatibility. However, limited research exists on wall deformations and the minimum achievable wall thickness between machined channels in PMMA via micro-milling. As microfluidic devices require tightly spaced features, identifying the minimum machinable wall thickness is essential for miniaturization and multifunctional integration, enabling rapid and reproducible biomedical testing. This study presents experimental data and finite element modeling on wall deformation characteristics—wall deviation angle, average wall thickness, and minimum machinable wall thickness—between micro-milled PMMA channels. Micro end-milling was performed with varying feed rates, wall thicknesses (50 μm, 100 μm, 150 μm), and milling strategies (direct, radial, axial depth). ANOVA was used to assess parameter influence, and finite element modeling simulated wall bending under the radial depth strategy. Results show that wall thickness, feed rate, and milling strategy significantly affect wall deviation and thickness. Experimental and simulation data revealed consistent trends: 50 μm walls showed cracking, base fractures, and geometric deviations, while 100 μm and 150 μm walls retained structural integrity. A minimum wall thickness of 150 μm is necessary to ensure reliable sealing in microfluidic devices.

## 1. Introduction

Micro-milling is widely applied in electronics, medical devices, aerospace, automotive, and jewelry industries [1]. Typical fabrication methods for microfluidic devices include lithography, embossing, injection molding, ion beam milling, and micro-milling. For small-scale production, micro-milling is advantageous as it eliminates tooling and pre-production steps required for mass manufacturing [2,3,4,5]. CNC programs can be prepared quickly, enabling immediate fabrication.

Compact microfluidic designs require minimal wall thickness between adjacent channels to maximize substrate utilization and allow rapid, reproducible biomedical testing. However, wall deformations—variations in thickness and taper angle—pose a critical challenge, as they affect flow behavior and device performance. Even minor deviations can alter split-flow conditions or daughter volume ratios [6,7,8,9]. Therefore, minimizing thickness and taper variations is essential for uniform fluid motion. Poly(methyl methacrylate) (PMMA) is commonly used for microfluidic devices due to its chemical resistance, low cost, optical transparency, and process compatibility.

Research on wall deformation and minimum machinable wall thickness in micro-milled substrates remains limited. Davoudinejad et al. [10] studied Al6082-T6 alloy using a 500 µm end-mill at 40,000 rpm, 8 µm/tooth feed, and 20 µm radial depth. FEM simulations revealed severe deformation in 50 µm walls, whereas 100 µm walls largely retained their shape, albeit with an 8.5% slope deviation near the top. Sun et al. [11] examined 75 µm thick walls on Al6061-T6; FEM and experimental results showed that the actual thickness exceeded the design value. Reduced stiffness at the wall top caused greater location errors, poorer surface quality, and increased concavity.

Literature on polymer micro-milling is scarce, and existing studies do not directly address channel wall deformation or minimum machinable wall thickness. However, several findings are noteworthy: Chris et al. [12] reported that polystyrene’s low elastic modulus and melting point complicate micro-milling. Goo et al. [13] showed that carbon nanotube reinforcement reduces plowing force and burr formation. Korkmaz et al. [14] analyzed the effects of spindle speed, feed rate, and axial depth on burrs, channel integrity, surface roughness, and cutting forces using PMMA channels with 150 µm walls and depths of 50–100 µm, machined by a 450 µm end-mill. None of these studies aimed to minimize wall thickness. Xue et al. [15] employed a rotating nano-indenter to machine PMMA channels of varying widths, thereby reducing the bottom surface roughness (Ra). Rodriguez et al. [16] optimized PMMA milling parameters, achieving $R_{a}=0.397\;\mu$m at 170 mm/min feed, 4238 rpm spindle speed, and 3.4 mm depth. Reichenbach et al. [17] investigated the effects of feed rate and cutting-edge geometry on tool wear using a 50 µm end-mill; a low feed per tooth (1 µm/tooth) improved surface finish but increased wear and burrs, whereas a higher feed (4 µm/tooth) extended tool life but worsened roughness. Jiao and Cheng [18] used a diamond ball end mill (1.5 mm diameter) for PMMA micro-milling; the slot strategy achieved a roughness of 8.7 nm Ra but failed on larger areas due to dynamic changes. Rahim et al. [19] fabricated PMMA channels (50 µm depth, 200 µm width, 1 cm length) using a 1 mm end-mill, finding that depth had the most significant impact on surface roughness, while feed rate had little influence. Meng et al. [20] compared strategies—down-feed, climb, and conventional milling—using a 1 mm end-mill at 80,000 rpm; exterior down-feed yielded better roughness. Yan et al. [21] linked surface quality to chip type and recommended processing below 70 °C. Later, Yan et al. [22] showed brittle removal at −55 °C and plastic removal at 25 °C, with roughness increasing with feed rate. Sun et al. [23] developed an FEM-based model for thermoset polymers; larger tool edge radii raised cutting forces and residual stresses, peaking at 36.4 MPa near the wall surface.

Current research on micro-milling primarily focuses on cutting force effects and their prediction using FEM and related techniques for metallic materials [24]. Demiral and Mamedov [25] applied a smoothed particle hydrodynamics (SPH)-based FEM model to predict cutting forces during Ti6Al4V milling with an 800 µm end-mill at 100 µm depth and 10,000 rpm; model predictions aligned well with experiments. Abeni et al. [26] proposed a 2D FEM approach for Inconel 625, using a 789 µm end-mill at varying feeds and speeds while maintaining a 200 µm wall thickness. FEM predictions matched analytical results, except during tool disengagement, where chip segmentation inaccuracies led to overestimation. Bhople et al. [27] developed an ABAQUS-based FEM model for Ti6Al4V to predict forces and von Mises stresses at 60,000 rpm, 50 µm depth, and feeds of 0.5–4 µm/tooth; results confirmed strong agreement with experiments. Material removal was dominated by plastic deformation at low feed rates, while higher feed rates increased tangential forces due to increased chip load.

Despite these advances, the literature lacks comprehensive experimental and theoretical studies on channel wall deformation under different machining strategies for polymeric materials, particularly PMMA. Most polymer studies emphasize surface quality rather than wall integrity. This work addresses this gap by examining the influence of milling strategies on wall deformation and determining the minimum machinable wall thickness between adjacent features. Specifically, the study evaluates wall bending, cracking, fracture, and thickness reduction in PMMA channels fabricated using a carbide end-mill under varying feed rates and three strategies: Direct Milling (DMS), Radial Milling (RDMS), and Axial Depth Milling (ADMS).

Finite Element Modeling (FEM) was employed to simulate the RDMS strategy, which exhibited a greater wall deviation angle compared to ADMS, in order to elucidate the underlying wall bending and deformation mechanisms. Finally, the minimum wall thickness required to seal a microfluidic device was determined using micro-reactors fabricated with wall thicknesses of 100 µm and 150 µm.

## 2. Materials and Methods

### 2.1. Experimental Study

**Figure 1:**
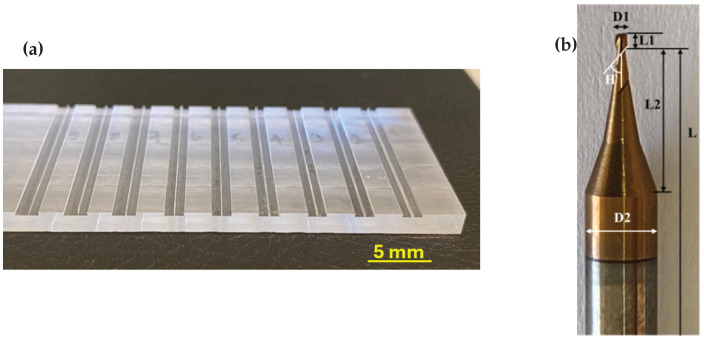
(**a**) A PMMA substrate with channels and walls. (**b**) A two-fluted micro end-mill.

The 400 µm deep channels and the 50 µm, 100 µm, and 150 µm thick walls between them on a 75 mm × 25 mm × 2.85 mm PMMA substrate (Figure 1a) were milled using a 693 µm diameter, 2-fluted tungsten carbide end-mill (Figure 1b). The properties of the PMMA material and the geometry of the end mill are provided in Table 1. The end mill was mounted on the ProLIGHT 1000 Machining Center (Light Machines Corp., Manchester, NH, USA) using a 4 mm ER20-4 GFB-4921 collet. In this study, each channel measured 25 mm in length. To micro-mill 18 walls of this length, the cutting tool completed 36 passes, resulting in a total machining length of 900 mm under full-diameter engagement conditions. This calculation neglects the finishing passes used in the RDMS and ADMS approaches, which involved minimal material removal. Due to the short machining length and the low hardness of PMMA, a single cutting tool was used for each set of experiments. No evidence of tool wear was detected on the cutting edges in the images captured at the conclusion of each test set. Consequently, the end mill was replaced upon completion of every experimental set.

The resolution of the machining center in the x, y, and z axes was 1 μm. According to the literature, it is recommended that the feed per tooth ($f_{z}$) be approximately equal to the cutting-edge radius of the tool [31,32,33,34] to minimize burr formation in metallic materials. The tool used in this study had a measured cutting-edge radius of 5 μm; therefore, a feed rate range of 4–7 μm/tooth would be reasonable for metals. However, no published data on $f_{z}$ settings for PMMA could be found.Nonetheless, due to PMMA’s low strength and rigidity, lower feed rate values are required than those used for metals. It is also essential that the end-milling process is sufficiently fast to ensure economic viability compared to other rapid fabrication techniques. For this reason, $f_{z}$ settings below 1.25 μm/tooth were not used in this study. Preliminary experiments showed that when $f_{z}$ exceeded 6 μm/tooth, the tool flutes could not effectively evacuate polymeric chips from the machining zone. Consequently, feed rates of 1.25, 3.13, and 5 μm/tooth were selected for the experiments.

A spindle speed (*n*) of 10,000 rpm was used to prevent thermally affected zones in the channel walls and bottoms, which were observed at higher speeds. The PMMA substrate was secured to the milling machine table using double-sided adhesive tape, as the end mill exerted only minimal force parallel to the table surface. Furthermore, a 0.1 MPa air jet—confirmed not to induce wall deflection during milling—was directed toward the machining zone through a nozzle with a 3 mm diameter opening, facilitating chip evacuation and maintaining cooling at the tool–substrate interface. Preliminary tests also showed that wall thicknesses (*t*) below 50 µm led to significant cracking and fracturing at the base of the walls due to excessive bending caused by the tool forces. Therefore, walls with thicknesses of 50 µm, 100 µm, and 150 µm were used in the experiments.

Each experiment was repeated twice to enhance the reliability of the results, and the average of the measurements was reported.

**Figure 2:**
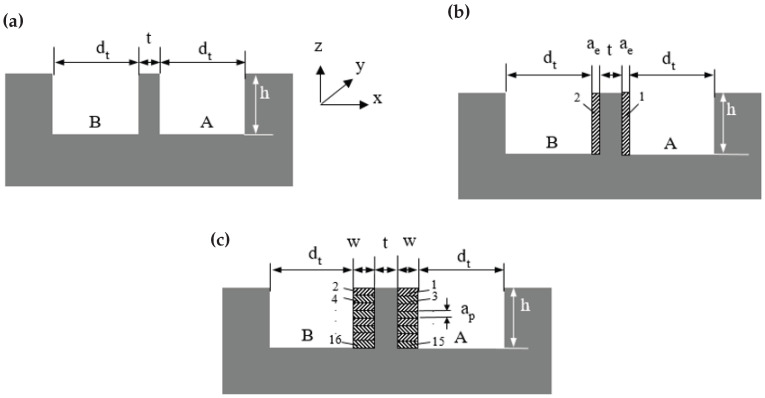
The micro-milling parameters and sequences of end-milling pass for (**a**) DMS, (**b**) RDMS, and (**c**) ADMS.

The operation sequences of DMS, RDMS, and ADMS are as follows:

Direct Milling Strategy (DMS)—Figure 2a

1. Channel A is milled to a width equal to the tool (end-mill) diameter $d_{t}$ and a depth *h*.
2. The tool exits Channel A by moving upward along the *z*-axis.
3. The tool mills Channel B to the same width ($d_{t}$) and depth (*h*), leaving a wall with thickness *t* between the channels.
4. The tool exits Channel B by moving upward along the *z*-axis.

Radial Milling Strategy (RDMS)—Figure 2b

1. Channel A is milled to a width equal to the tool diameter $d_{t}$ and a depth *h*.
2. The tool exits Channel A by moving upward along the *z*-axis.
3. Channel B is milled to the same width and depth, leaving a wall with a thickness of $t+2a_{e}$, where $a_{e}$ is the radial depth of cut.
4. The tool exits Channel B by moving upward along the *z*-axis.
5. The tool is fully inserted into Channel A.
6. The left wall of Channel A is milled with a radial depth of cut $a_{e}$.
7. The tool exits Channel A by moving upward along the *z*-axis.
8. The tool is fully inserted into Channel B.
9. The right wall of Channel B is milled with a radial depth of cut $a_{e}$.

The radial depth of cut ($a_{e}$), also known as the stepover, depends on the wall material, required geometric and dimensional tolerances, and surface roughness in finishing applications. In industrial practice, stepover values typically range from 3% to 15% of the tool diameter for finishing passes [35]. Accordingly, $a_{e}$ was set to 50μm (approximately 7% of $d_{t}$) in this study.

Axial Depth Milling Strategy (ADMS, Step-Down Approach)—Figure 2c.

In ADMS, the wall (or channel) height *h* is divided into smaller axial depths, designated as the axial depth of cut $a_{p}$. In Figure 2c, the dashed areas (#1–#16) each have a height of $a_{p}$ and a width *w* (cutting width). ADMS provides improved wall support compared to DMS and RDMS, particularly for thin-wall milling.

1. Channel A is milled to a width equal to the tool diameter $d_{t}$ and a depth *h*.
2. The tool exits Channel A by moving upward along the *z*-axis.
3. Channel B is milled to the same width and depth, leaving a wall with a thickness of t+2w between the channels.
4. The tool exits Channel B by moving upward along the *z*-axis.
5. The tool partially enters Channel A and removes area #1 (depth $a_{p}$, width *w*) using axial (downward) feed.
6. The tool exits Channel A by moving upward along the *z*-axis.
7. The tool partially enters Channel B and removes area #2 (depth $a_{p}$, width *w*) using axial (downward) feed.
8. The tool exits Channel B by moving upward along the *z*-axis.
9. Steps 5 to 8 are repeated until all dashed areas are removed.
10. Finally, the tool exits Channel B by moving upward along the *z*-axis.

The machining parameters ($a_{p}$, $a_{e}$, *w*, and *h*) used in the experiments are listed in Table 2. In this study, $a_{e}$ was set equal to $a_{p}$, and *w* was set equal to *h*, to maintain a constant cross-sectional cutting area for RDMS ($a_{e}\times h$) and ADMS ($a_{p}\times w$) strategies. This approach ensured constant cutting power across all experiments, regardless of the strategy employed.

All walls were milled using the down-milling technique to minimize the tool forces acting on the walls. The Taguchi experimental design method was used with an L27 orthogonal array. The parameter settings are provided in Table 3.

All channel and wall side-view images were captured using a Nikon Eclipse LV150N microscope (Tokyo, Japan) and processed with the open-source ImageJ software 1.53a. Due to the tapered geometry of the machined walls, where the top thickness exceeded that of the bottom, conventional surface characterization techniques such as profilometry and confocal microscopy were not applicable.

**Figure 3:**
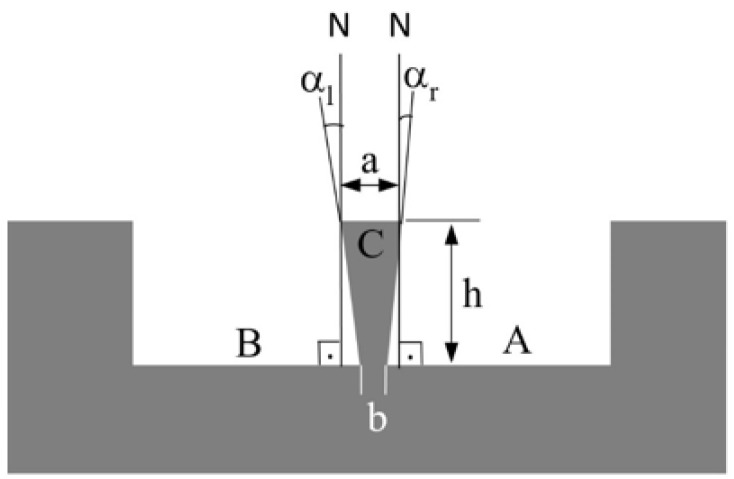
Schematic presentation of the channels (A and B), the wall, and the left and right-side wall deviation angles ($\alpha_{l}$ and $\alpha_{r}$).

Two metrics were defined in this study to analyze the effects of the set wall thickness *t* and milling strategy on wall geometry. The first metric is the average wall thickness ($t_{\mathrm{av}}$), calculated as the average of the wall top thickness (*a*), wall bottom thickness (*b*), and the thickness at the midpoint of wall height (Figure 3). This metric indicates how closely the micro-milled wall thickness matches the set value *t*. The second metric is the wall deviation angle (α), defined as the sum of the angles between the wall’s side surfaces and the normal (*N*) to the channel bottom surface, i.e., the sum of the left-side angle ($\alpha_{l}$) and right-side angle ($\alpha_{r}$). This metric indicates the deviation of the wall geometry from the ideal rectangular form (i.e., wall conicity).

### 2.2. Numerical Simulation Procedure

**Figure 4:**
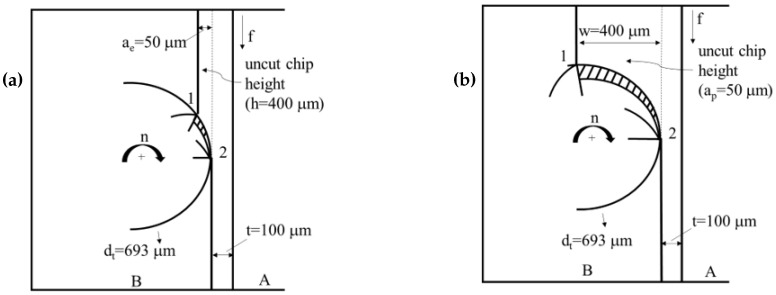
Top view of chip formation for (**a**) RDMS and (**b**) ADMS.

The magnitudes of the cutting force components acting on the wall differ between RDMS and ADMS. Although the same amount of material is removed per pass, ensuring constant cutting power during milling, a shorter uncut chip is removed by each cutting edge in RDMS compared to ADMS (Figure 4). Analytical approaches for calculating shear stresses, normal stresses, bending moments, and cutting force components acting on the wall are highly complex, as these factors influence wall bending and the formation of wall angles. Although experimental studies were conducted in this work, micro-milling operations remain inherently challenging, and accurately measuring micro-milled forms—particularly deviations of walls from their intended geometries—remains difficult. To complement the experiments and provide guidance for future research, this study also employs finite element modeling (FEM) of the micro-milling process. The goal is to assess whether FEM can accurately approximate wall deformations without requiring highly complex experiments, thereby providing a practical reference for future investigations.

FEM was employed to estimate the theoretical wall deviation angle values at t=100μm and $f_{z}=1.25$, 3.13, and 5μm/tooth for the RDMS strategy. RDMS was selected for this analysis since it produces a greater wall deviation angle (α) than ADMS. Moreover, the von Mises equivalent stress values along the 400μm wall height were determined by sampling discrete points at uniform intervals of 10μm from the stress distribution obtained through the FEM analysis. This procedure enabled a high-resolution assessment of stress variation across the specified height, ensuring accurate representation of localized stress concentrations.

ANSYS 2020R2 was used to develop a numerical model simulating the micro-milling process. SOLID185 elements (eight-node, three degrees of freedom) and Tet4 elements (four-node tetrahedral) were used to mesh the PMMA substrate and cutting tool, respectively. Mesh dependency tests showed that approximately 39,000 elements for the substrate and 91,000 elements for the cutting tool were sufficient to produce reliable results. The average mesh quality was 0.90.

#### 2.2.1. Boundary Conditions

**Figure 5:**
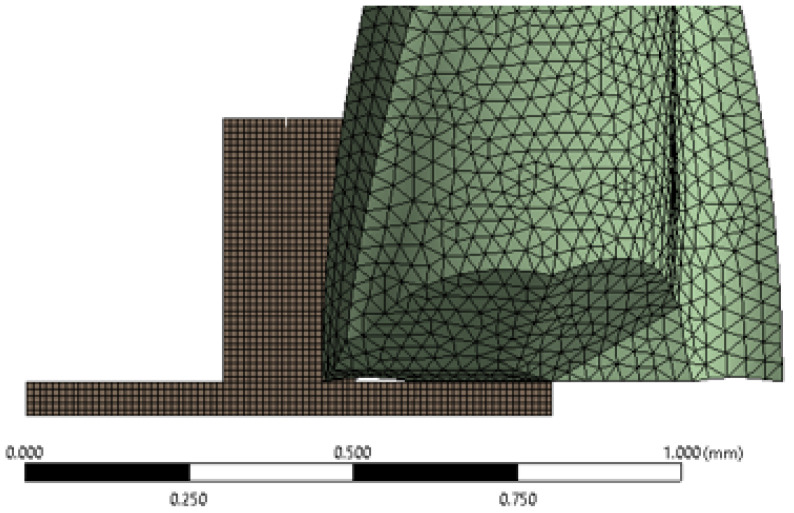
The reduced-size FEM of the PMMA substrate and the cutting tool.

A reduced-size model (Figure 5) was employed to represent the substrate, as preliminary analyses indicated no significant difference in wall deviation angle values between the full-size model (30 mm wall length and 700 μm channel width) and the reduced-size model (700 μm wall length and 350 μm channel width). Deformations along the wall length were assumed to be negligible because the wall length was substantially greater than its thickness (*t*) and height (*h*). The following conditions were applied:

- An end-mill spindle speed of 10,000 rpm was applied.
- A friction coefficient of 0.3 was specified between the PMMA substrate and the end mill.
- Feed rates for the PMMA workpiece were set to 1.25, 3.13, and 5 μm/tooth.
- All rotational degrees of freedom were permitted.
- Thermal load information and damping parameters were not taken into account.
- The cutting tool was modeled as a rigid body, whereas the PMMA substrate was modeled as a deformable body.

#### 2.2.2. Input Parameters

Material properties of the PMMA substrate included Young’s modulus, yield strength (Table 1), Poisson’s ratio, and the plastic stress–strain relationship. The true stress–true strain data at 20 °C and a strain rate of 1 s^−1^—the highest strain rate reported in the literature for PMMA [32]—were adopted, as high strain rates were anticipated and no melting or thermally affected zones were observed on the milled surfaces. The elastic–J2 (von Mises) plasticity [36] approach can be applied to amorphous polymers in forming and machining simulations with low error; therefore, J2 plasticity can be adopted as the fundamental elasto-plastic framework for PMMA milling simulations [37,38,39].

The following parameters were implemented in the numerical model:

- A frictional contact condition with a coefficient of friction of 0.3.
- End-mill spindle speed of 10,000 rpm.
- Feed velocities of the PMMA substrate in the x- and y-directions were set to 2.5, 6.25, and 10 mm/min, corresponding to feed rates of 1.25, 3.13, and 5 μm/tooth.

## 3. Results

### 3.1. Wall Geometric Characteristics

**Figure 6:**
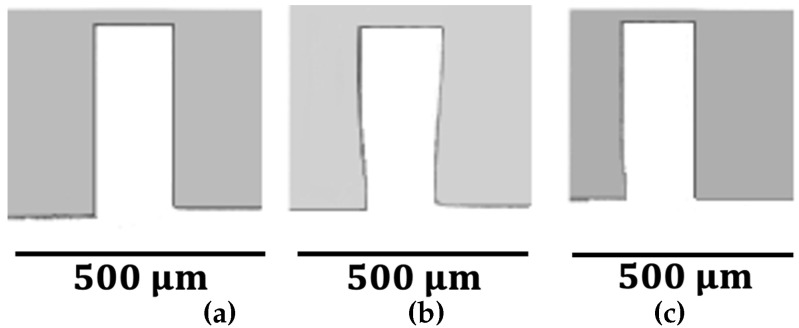
Images of t=150μm walls (side views). (**a**) $f_{z}=1.25\;\mu$m, DMS; (**b**) $f_{z}=3.13\;\mu$m, RDMS; (**c**) $f_{z}=5\;\mu$m, ADMS.

**Figure 7:**
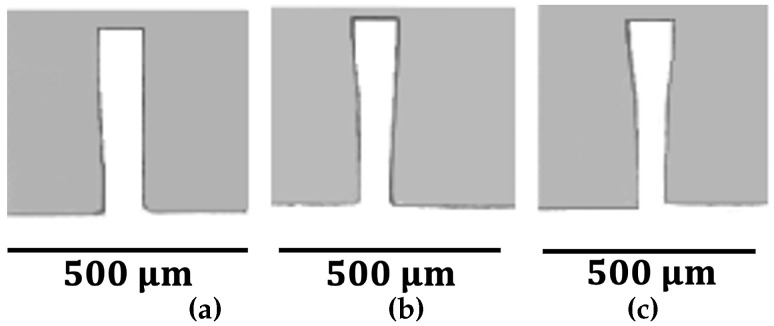
Images of t=100μm walls (side views). (**a**) $f_{z}=1.25\;\mu$m, ADMS; (**b**) $f_{z}=3.13\;\mu$m, ADMS; (**c**) $f_{z}=5\;\mu$m, ADMS.

**Figure 8:**
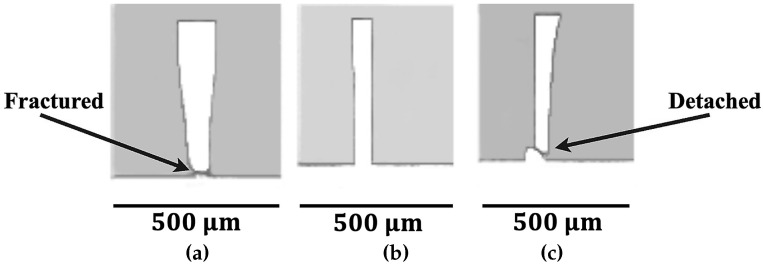
Images of t=50μm walls (side views). (**a**) $f_{z}=1.25\;\mu$m, RDMS; (**b**) $f_{z}=3.13\;\mu$m, ADMS; (**c**) $f_{z}=5\;\mu$m, ADMS.

Sample wall images from the milled specimens are presented in Figure 6, Figure 7 and Figure 8. The side-view images shown in these figures were captured directly from the inlet region where the end-mill entered the PMMA substrate, as cutting off the machined specimens was not feasible due to the thin walls. The micro-milled channel widths in DMS ranged between 699 and 704 μm. The outer sidewalls of the channels, as well as the left sidewalls of channel A, were nearly perpendicular to the channel bottoms. Burr formation at the tops of the walls was rare for all strategies, DMS, RDMS, and ADMS.

The 150 μm thick walls closely resembled a rectangular form (Figure 6), while the 100 μm thick walls showed slight deviations (Figure 7). Short bottom cracks were occasionally observed in the 100 μm thick walls near the tool entrance and exit regions, which could be mitigated using a lower $f_{z}$ value. In contrast, the 50 μm thick-walls exhibited an apparent deviation from the rectangular shape (Figure 8) and were frequently fractured or detached at the bottom near the tool entrance and exit areas. However, continuous cracks along the entire wall bottom were rare.

The 50 μm- and 100 μm thick walls, regardless of the milling strategy, exhibited a greater top wall thickness (*a*) than bottom wall thickness (*b*). However, the *b* values were close to the set wall thickness *t*. This discrepancy between *a* and *b* is attributed to wall bending. The cutter applies force to the wall during milling, causing it to bend. The upper portion of the wall displaces more than the lower region due to its greater distance from the substrate base. As a result, less material is removed from the top portion of the wall, leading to a thicker top and thinner bottom. Due to their higher stiffness, this effect was less significant in the 150 μm thick walls.

**Figure 9:**
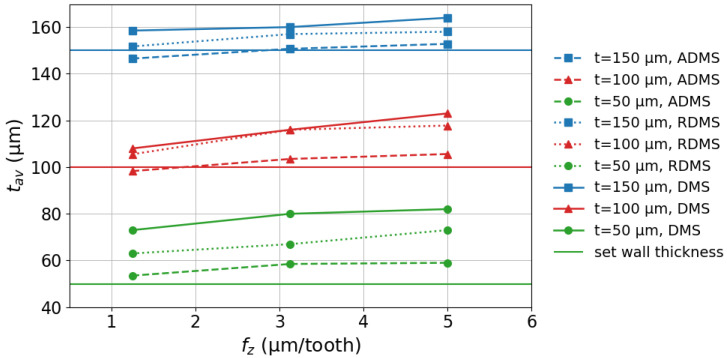
Effect of $f_{z}$, *t*, and milling strategies on $t_{\mathrm{av}}$.

As shown in Figure 9, the difference between the average wall thickness $t_{\mathrm{av}}$ and the set wall thickness *t* increased as *t* decreased. For the $f_{z}$ settings and milling strategies used in this study, the deviation between $t_{\mathrm{av}}$ and *t* ranged from −2.3% to +9.3% for t=150μm. For t=100μm, the deviation ranged from −23% to +1.7%, and for t=50μm, from +6% to +64%. In all cases, $t_{\mathrm{av}}$ was greater than *t*, and wall bending increased with decreasing *t*. Figure 9 also shows that higher $f_{z}$ values slightly increased the deviation between $t_{\mathrm{av}}$ and *t* due to the increased cutting forces acting on the walls, leading to greater bending.

Among the strategies, ADMS produced $t_{\mathrm{av}}$ values closest to the set thickness *t*. RDMS also performed better than DMS in this regard. Therefore, it can be concluded that the ADMS and RDMS strategies are more effective than DMS in minimizing the deviation between $t_{\mathrm{av}}$ and *t*.

**Figure 10:**
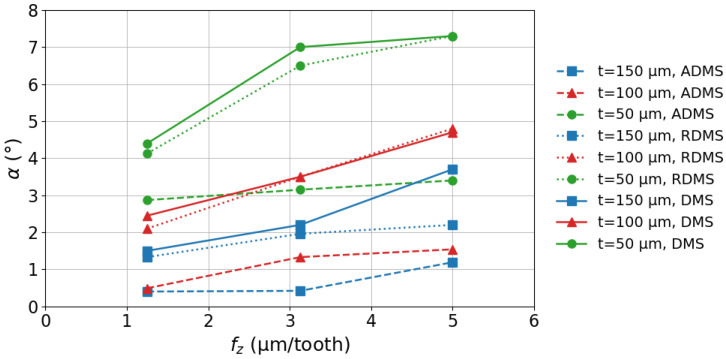
Effect of $f_{z}$, *t*, and milling strategies on α.

The effects of feed rate, set wall thickness, and milling strategies on the wall deviation angle α are shown in Figure 10. The following key observations can be made: The value of α increases as the wall thickness *t* decreases. For walls with thicknesses of 150 μm, 100 μm, and 50 μm, and 50 μm, α ranges from 0.4° to 3.7°, 0.5° to 4.8°, and 2.9° to 7.3°, respectively. The elevated α values observed at higher $f_{z}$ settings are attributed to increased cutting forces acting on the walls, which lead to greater bending. Among the strategies, ADMS resulted in the smallest α, while RDMS showed the second-lowest values.

The standard deviations of $t_{\mathrm{av}}$ values in Figure 9 range from 1.6% to 2.4% for t=50μm, 2.0% to 2.8% for t=100μm, and 2.4% to 3.1% for t=150μm, depending on the $f_{z}$ settings. Similarly, the standard deviations of α values in Figure 10 range from 0.7% to 4.2% for all thicknesses (t=50μm, 100μm, and 150μm), also depending on the $f_{z}$ settings. Since the standard deviations of $t_{\mathrm{av}}$ and α are relatively small, the observed effects can be considered significant compared to the experimental scatter.

### 3.2. Statistical Analysis

In this study, the above-mentioned experimental findings about wall geometrical characteristics were compared with findings of the loss function in the Taguchi method. The Taguchi method used a loss function to calculate the deviation between experimental and desired values. This loss function is then transformed into a signal-to-noise (S/N) ratio. In this study, low values of α and $t_{\mathrm{av}}$ indicate better performance. Therefore, a lower-is-better (LB) S/N ratio was selected for both performance outputs to obtain optimum machining performance characteristics. Irrespective of the performance characteristics category, a higher S/N value indicates better performance. Thus, the highest S/N value represents the optimal level of the process parameter. The S/N values for each experiment presented in Table 4 and Table 5 revealed that the optimal performances for α and $t_{\mathrm{av}}$ were achieved at level 1 for the $f_{z}$ (1.25 μm/tooth), at level 3 for the milling strategy MS (ADMS), and level 1 for the *t* (150 μm). As shown above, the S/N ratio analysis and the experimental results are in agreement.

In this study, analysis of variance (ANOVA) was employed to determine the extent to which process parameters influence performance outputs, specifically the wall deviation angle (α) and the average wall thickness ($t_{\mathrm{av}}$), as presented in Table 6 and Table 7. The statistical significance of the observed differences between group means was assessed by examining the corresponding *p*-values for the *F*-statistics obtained from the ANOVA results. If p<0.05, it is concluded that there is a statistically significant difference between the group means. Since the *p*-values in both tables are less than 0.05, the differences between the group means are statistically significant. Table 8, which summarizes Table 6 and Table 7, shows that the primary factor influencing α and $t_{\mathrm{av}}$ was the wall thickness (*t*), contributing 45.29% and 75.24%, respectively. The ADMS milling strategy ranked second, contributing 27.03% to α and 17.30% to $t_{\mathrm{av}}$. In contrast, the feed per tooth ($f_{z}$) exhibited the least influence on variations in α and $t_{\mathrm{av}}$, with respective contributions of 12.29% and 1.85%.

### 3.3. Microfluidic Device Production

The cross-sectional wall images (Figure 8) revealed that the 50 μm thick walls were unsuitable for microfluidic device applications due to high wall deviation angles, fractures and cracks at the wall bottoms. Moreover, these 50 μm thick walls may bend, crack, or fracture entirely under the compressive force applied to the micro-milled substrate during the sealing (bonding) of the microfluidic device.

**Figure 11:**
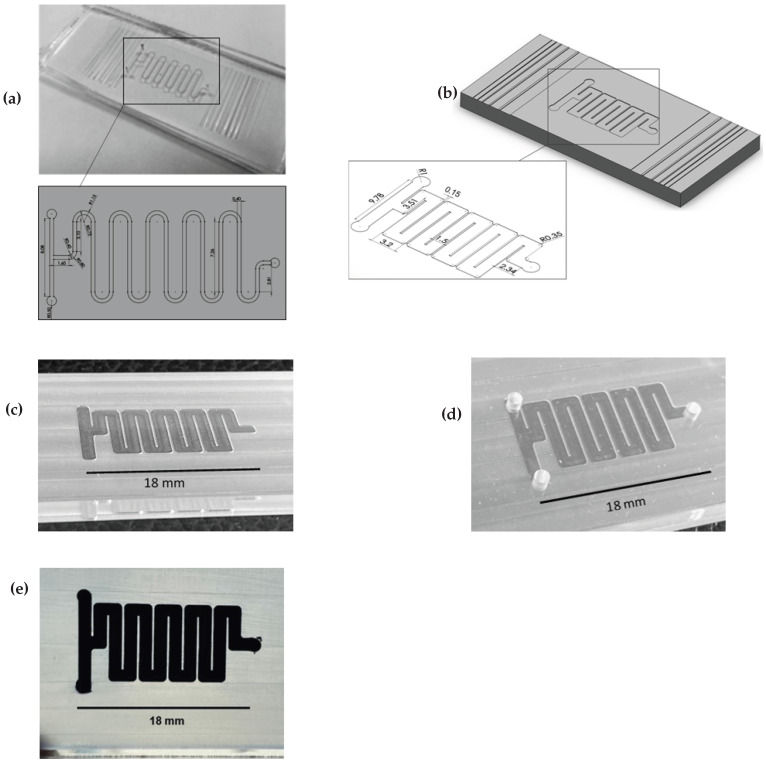
Images of the micro-reactor: (**a**) the micro-reactor produced by hot embossing in Çoğun et al. [28] study (dimensions in mm; channel depth *h* is 122 μm); (**b**) the micro-reactor design in the present study (dimensions in mm); (**c**) after milling; (**d**) after bonding; (**e**) after leakage test.

To determine the minimum wall thickness required for successful sealing, sets of microfluidic devices (micro-reactors) were fabricated with 100 μm and 150 μm thick walls at a depth of h=400μm, with five devices produced for each thickness value. Sealing performance was evaluated using a leakage test. In this test, two drops of black food dye diluted 50% with distilled water and pure distilled water were simultaneously introduced into the two inlet ports of the micro-reactor using separate pipettes. Since the micro-reactor is a passive type, fluid flow within the device channels occurred naturally (i.e., no pressure was applied to the ports), and successful mixing was achieved within 6 s when the mixed fluid reached the exit port of the device. The leakage test was considered failed if the dye–water mixture leaked between the top of the device walls and a 1 mm thick PMMA sealing substrate, or if fluid flow stopped before reaching the exit port. For failed micro-reactors, the wall bottoms and tops were not micrographed, as the walls were destroyed during the removal of the sealing substrate. The micro-reactor had the same channel and port design as the one produced by hot embossing in the study by Çoğun et al. [28], although the channel width was increased to 1500 μm to enable the use of ADMS for wall fabrication with a $d_{t}=693\;\mu m$ end-mill (Figure 11b).

A 1 mm thick blank PMMA substrate was used to seal the micro-milled channels under bonding parameters of 3 kN force, 3 min duration, 85 °C temperature, and 4 min of chloroform vapor treatment prior to bonding. In this study, the micro-reactor walls with a thickness of 100 μm failed the leakage test due to cracks and fractures caused by the compressive bonding force. In contrast, the micro-reactors with 150 μm thick walls (Figure 11b–e) passed the leakage test successfully.

This study demonstrated that for PMMA material machined using micromilling, a minimum wall thickness of 150 μm is required for reliable compressive bonding to seal a microfluidic device. In comparison, 100 μm walls are suitable for microdevices that do not require compressive sealing during fabrication, such as microactuators, microoptics, transistors, microcircuits, or wafers.

In the micro-milling of devices with 150 μm thick walls, the lowest feed rate ($f_{x}=1.25\;\mu m/$tooth) and the optimal milling strategy (ADMS) were employed to achieve the best geometric wall profiles with minimum α and $t_{\mathrm{av}}$ (Figure 9 and Figure 10). However, measurements taken after machining the micro-reactor walls revealed that the values of α and $t_{\mathrm{av}}$ differed from those shown in Figure 9 and Figure 10, with $t_{\mathrm{av}}$ approximately 5% higher and α approximately 8% higher, due to changes in the cutting tool feed direction during wall machining. Machining the two sides of a wall in the reverse direction bends the micro-reactor’s walls more, thereby increasing both the average wall thickness and the wall taper angle.

The micro-reactor occupied approximately 25% less space than the one referenced in the literature, simply by reducing the thickness of the walls between the channels (Figure 11b), despite using channels that were 1100 μm wider than those in Reichenbach et al. [17]. The space savings would have exceeded 35% if a 400 μm-diameter end-mill had been used to fabricate channels with an 800 μm width.

### 3.4. Numerical Simulation Results

**Figure 12:**
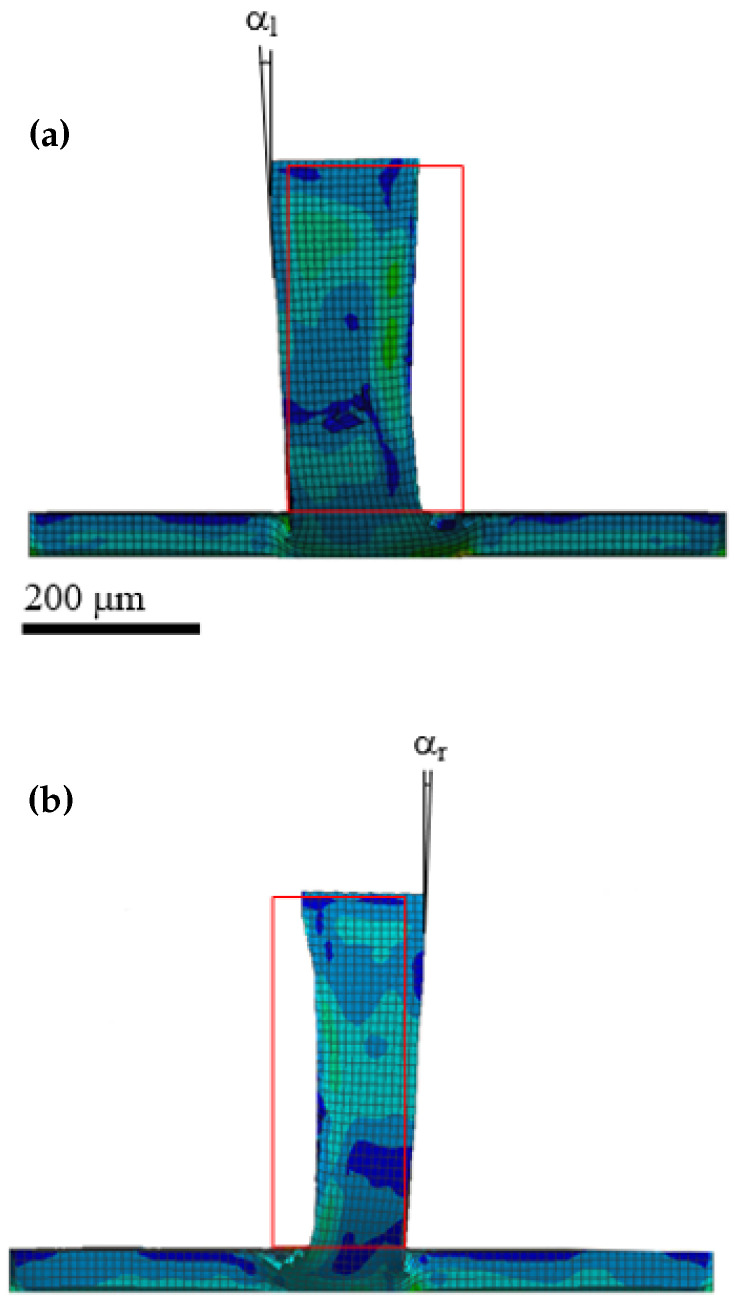
The FEM images showing wall bending and the α. After machining (**a**) the right side of the wall and (**b**) the left side of the wall (the red rectangles show the unmachined wall form).

In this study, FEM was employed to determine the theoretical values of the wall deviation angle (α) at t=100μm and $f_{z}=1.25$, 3.13, and 5μm/tooth. In the model, the initial wall thickness of 200μm was reduced to the target thickness of 100μm using the RDMS approach. The FEM results for the right and left sides of the walls are presented in Figure 12. The red-outlined rectangles represent the wall shape before milling. The angles between the wall side surfaces and the channel bottom surface normal ($\alpha_{l}$ and $\alpha_{r}$), resulting from wall bending, are visible.

**Figure 13:**
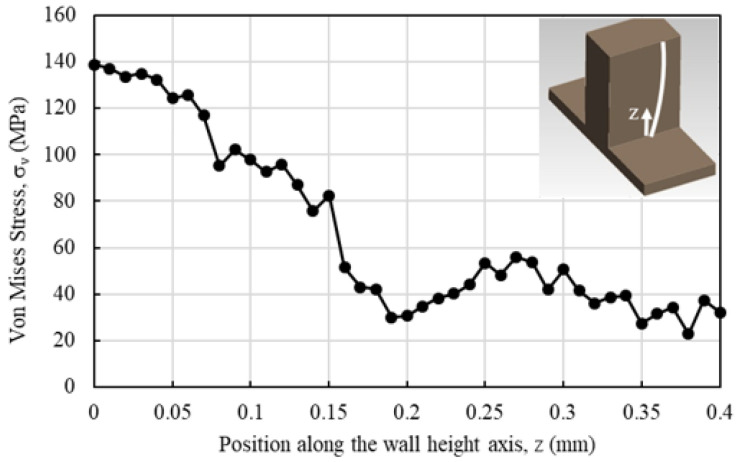
The variation of $\sigma_{v}$ along the wall height axis *z*.

The Von Mises stress ($\sigma_{v}$) distribution along the wall in the contact region of the cutting edge (Figure 13) showed a decreasing trend along the wall height (*z*-axis). The observed reduction occurs because wall bending leads to less material removal (i.e., smaller uncut chip thickness) in the upper sections of the wall. At the upper sections, the $\sigma_{v}$ value was approximately 30 MPa, whereas at the bottom wall (z=0), it increased to 140 MPa. This stress level exceeds the fracture strength of the PMMA substrate, leading to local crack and fracture formation. The higher $\sigma_{v}$ values near the wall base are attributed to increased lateral stiffness, which resists the lateral motion of the end mill.

**Figure 14:**
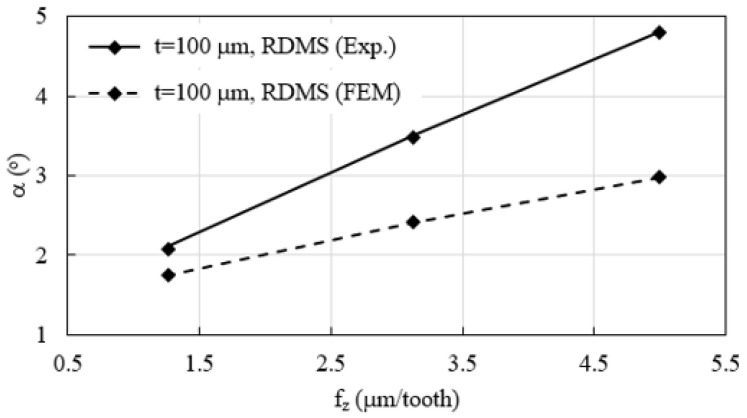
The variation of α with $f_{z}$ for experimental and FEM results (t=100μm, RDMS).

The FEM analysis corroborated the experimental findings, demonstrating that an increase in $f_{z}$ leads to an increase in α (Figure 14). The experimentally measured α values were 17–38% higher than the FEM predictions across the tested $f_{z}$ settings. This discrepancy is expected, as the study contributes to the early efforts in modeling the micro-milling process of a polymeric substrate (PMMA) using FEM. Several factors may contribute to this divergence, including uncertainties in the PMMA stress–strain data and thermal softening characteristics, omission of viscoelastic/viscoplastic behavior of PMMA, approximations related to edge rounding and lateral deflection of the end mill, and simplifying assumptions adopted in the FEM model.

## 4. Conclusions

This study examines channel wall deformation in PMMA substrates by analyzing wall deviation angles and average wall thickness under three distinct milling strategies: Direct Milling (DMS), Radial Milling (RDMS), and Axial Depth Milling (ADMS). The findings establish the minimum wall thickness required to ensure leak-free performance in microfluidic devices, thereby providing critical design benchmarks for researchers developing miniaturized polymer-based systems.

The results revealed that the wall-top thickness exceeded the wall-bottom thickness for 50 μm and 100 μm thick walls due to tool-induced bending, whereas the 150 μm thick walls exhibited minimal variation. As the wall thickness decreased, the wall deviation angle increased, and the average wall thickness deviated further from the set value. Higher feed rates intensified these effects, resulting in reduced average wall thickness and increased deviation angles. ADMS yielded the most accurate wall geometries among the tested strategies with minimal dimensional deviations.

Taguchi S/N ratio analysis indicated that combining the lowest feed rate, highest wall thickness, and ADMS settings produced the most favorable outcomes, minimizing wall deviation and aligning average wall thickness closely with design specifications. Analysis of Variance (ANOVA) confirmed that wall thickness had the most significant influence on both metrics, while feed rate had the least significant influence.

Finite element modeling supported the experimental findings, with differences ranging from 17% to 38%. This analysis revealed elevated Von Mises stresses near the base of thin walls, which contributed to cracking, fracturing, and increased deviation under higher feed rates. A minimum wall thickness of 150 μm was identified as necessary for reliable sealing of microfluidic devices, although 100 μm thick walls were also acceptable in terms of structural integrity and geometry. The minimum wall thickness threshold of 150 μm applies specifically to the tested tool diameter, feed rate range, and the solvent-assisted thermal bonding protocol. Variations in tool geometry, machining parameters, or bonding methodology may alter this threshold. The micro-reactor fabricated in this study was notably smaller than previously reported designs, underscoring the potential for further miniaturization. When the variation of α with $f_{z}$ between the experimental and FEM results is compared, a noticeable difference is observed. To reduce this discrepancy, a more accurate description of the material behavior is expected to be provided by the incorporation of a viscoplastic constitutive model in future work, thereby effectively resolving the observed inconsistency.

A smaller wall deviation angle and reduced wall thickness could potentially be achieved specifically for PMMA by using feed rates below 1.25 μm/tooth, spindle speeds above 10,000 rpm with mist cooling, and channel depths (i.e., wall heights) of less than 400 μm, as implemented during the experimental phase of this study. However, these settings result in longer machining times, deeper thermally affected zones in the walls, and insufficient channel depth for steady fluid flow in microfluidic devices.

Future research should investigate the mechanical behavior of thin-walled PMMA structures under compressive loads and exposure to sealing agents, such as chloroform vapor, to further validate the integrity of the walls.

## Figures and Tables

**Table 1:** Properties of PMMA [28,29,30] and Geometry of Cutting Tool (Figure 1).

**PMMA**	
Grade	8560K196
Manufacturer	McMaster-Carr (Elmhurst, IL, USA)
Production Technique	Emulsion Polymerization
Molar Mass	Variable
Density at 30 °C (g/cm^3^)	1.185
UTS at 20 °C (MPa)	130
Yield Strength at 20 °C (MPa)	95
Elastic modulus at 30 °C (MPa)	5750
Melting temperature (°C)	160
Glass transition temperature (°C)	107
Hardness (Shore D)	90
Thermal conductivity (W/m·K)	0.18
**Cutting Tool**	
Manufacturer	SUNVO TOOLS CORP. (Jiangyin, China)
Material	WC Tip, Steel Shank
Cutting Edge Diameter, D_1_ (µm)	693
Helix Angle, H (°)	30
Number of Flutes	2 (L_1_ = 1.4 mm)
Shank Diameter, D_2_ (mm)	4
Rake Angle (°)	8
Cutting Edge Radius (µm)	5
Surface Coating	Uncoated
Shank Conical Length, L_2_ (mm)	8.5
Tool Overall Length, L (mm)	50
Hardness (HRC)	55

**Table 2:** Micro-milling parameters for wall fabrication.

Set Wall Thickness *t* (µm)	Tool Diameter $d_{t}$ (µm)	Wall Height *h* (µm)	Radial Depth of Cut $a_{e}$ (µm)	Axial Depth of Cut $a_{p}$ (µm)	Width of Cut *w* (µm)
150 100 50	693	400	50	50	400

**Table 3:** Experimental design.

Parameters	Settings
Feed per tooth $f_{z}$ (μm/tooth)	1.25, 3.13, 5
Milling strategy	DMS, RDMS, ADMS
Set wall thickness *t* (μm)	50, 100, 150

**Table 4:** Response table for S/N ratios for the α.

Level	$f_{z}$	MS	*t*
1	−8.308	−13.716	−6.522
2	−11.344	−13.574	−10.300
3	−13.432	−5.794	−16.263

**Table 5:** Response table for S/N ratios for the $t_{\mathrm{av}}$.

Level	$f_{z}$	MS	*t*
1	−40.62	−42.03	−38.51
2	−40.99	−41.69	−40.86
3	−41.47	−39.36	−43.71

**Table 6:** Result of ANOVA for α.

Source	DF	Seq SS	Contribution	Adj SS	Adj MS	F-Value	*p*-Value
$f_{z}$	2	25.18	12.29%	25.18	12.588	7.99	0.003
MS	2	55.35	27.03%	55.35	27.674	17.56	0.000
*t*	2	92.76	45.29%	92.76	46.381	29.44	0.000
Error	20	31.51	15.39%	31.51	1.576		
Total	26	204.80	100.00%				

**Table 7:** Result of ANOVA for $t_{\mathrm{av}}$.

Source	DF	Seq SS	Contribution	Adj SS	Adj MS	F-Value	*p*-Value
$f_{z}$	2	503.2	1.85%	503.2	251.6	3.30	0.050
MS	2	4708.8	17.30%	4708.8	2354.4	30.87	0.000
*t*	2	20,474.1	75.24%	20,474.1	10,237.0	134.22	0.000
Error	20	1525.5	5.61%	1525.5	76.3		
Total	26	27,211.6	100.00%				

**Table 8:** ANOVA Summary of the Effects of α and $t_{av}$ on t, MS, and $f_{z}$.

Source	α			$t_{\mathrm{av}}$		
	F-Value	*p*-Value	Contribution (%)	F-Value	*p*-Value	Contribution (%)
t	29.44	0.000	45.29	134.22	0.000	75.24
MS	17.56	0.000	27.03	30.87	0.000	17.30
$f_{z}$	7.99	0.003	12.29	3.30	0.050	1.85
Error	–	–	15.39	–	–	5.61

## Data Availability

The original contributions presented in this study are included in the article. Further inquiries can be directed to the corresponding author.

## Abbreviations

The following abbreviations are used in this manuscript:

ADMS	Axial Depth Milling Strategy
DMS	Direct Milling Strategy
FEM	Finite Element Modeling
RDMS	Radial Drilling Strategy
*a*	Wall top thickness (μm)
$a_{r}$	Radial depth of cut (μm)
$a_{z}$	Axial depth of cut (μm)
*b*	Wall bottom thickness (μm)
$d_{t}$	Cutting tool diameter (μm)
$f_{z}$	Feed per tooth (μm/tooth)
*h*	Channel depth and wall height (μm)
*n*	Cutting tool spindle speed (rev/min)
*w*	Width of cut (μm)
*t*	Set wall thickness (μm)
$t_{d}v$	Average wall thickness (μm)
α	Wall deviation angle (°)
$\alpha_{l}$	Wall left side deviation angle (°)
$\alpha_{r}$	Wall right side deviation angle (°)

## References

[1] O’Toole L., Kang C.W., Fang F.Z. (2021). Precision micro-milling process: State of the art. Adv. Manuf..

[2] Bediz B., Korkmaz E., Khilwani R., Donahue C., Erdos G., Falo L.D.J., Ozdoganlar O.B. (2014). Dissolvable microneedle arrays for intradermal delivery of biologics: Fabrication and application. Pharm. Res..

[3] Korkmaz E., Friedrich E.E., Ramadan M.H., Erdos G., Mathers A.R., Ozdoganlar O.B., Washburn N.R., Falo L.D.J. (2015). Therapeutic intradermal delivery of tumor necrosis factor alpha antibodies using tip-loaded dissolvable microneedle arrays. Acta Biomater..

[4] Wilson M.E., Kota N., Kim Y.T., Wang Y., Stolz D.B., LeDuc P.R., Ozdoganlar O.B. (2011). Fabrication of circular microfluidic channels by combining mechanical micro-milling and soft lithography. Lab Chip.

[5] Şimşek Ü., Davut K., Miyamoto H., Yalçınkaya T. (2024). Comparison of Linear and Nonlinear Twist Extrusion Processes with Crystal Plasticity Finite Element Analysis. Materials.

[6] Link D.R., Anna S.L., Weitz D.A., Stone H.A. (2004). Geometrically mediated breakup of drops in microfluidic devices. Phys. Rev. Lett..

[7] Garstecki P., Fuerstman M.J., Stone H.A., Whitesides G.M. (2006). Formation of droplets and bubbles in a microfluidic T-junction—Scaling and mechanism of break-up. Lab Chip.

[8] Agnihotri S.N., Raveshi M.R., Bhardwaj R., Neild A. (2019). Droplet breakup at the entrance to a bypass channel in a microfluidic system. Phys. Rev. Appl..

[9] Raveshi M.R., Agnihotri S.N., Bhardwaj R., Neild A. (2019). Selective droplet splitting using single-layer microfluidic valves. Sens. Actuators B Chem..

[10] Davoudinejad A., Li D., Zhang Y., Tosello G. Simulation of thin features machining by micro end milling using finite element modelling. Proceedings of the 19th International Conference and Exhibition (EUSPEN 2019).

[11] Sun Q., Zhou J., Li P. (2022). Simulations and Experiments on the Micro-Milling Process of a Thin-Walled Structure of Al6061-T6. Materials.

[12] Christ K.V., Smith B.B., Pfefferkorn F.E., Turner K.T. Micro end milling polystyrene for microfluidic applications. Proceedings of the 5th International Conference on Micromanufacturing.

[13] Goo C., Menard M., Huynh L.H., Papadopoulos C., Park S.S., Jun M.B.G. Micromachining of polymer composites reinforced with single-walled carbon nanotubes. Proceedings of the 5th International Conference on Micromanufacturing.

[14] Korkmaz E., Önler R., Özdoğanlar O.B. (2017). Micromilling of Polymethyl methacrylate (PMMA) using single crystal diamond tools. Procedia Manuf..

[15] Xue B., Geng Y., Yan Y., Ma G., Wang D., He Y. (2020). Rapid prototyping of microfluidic chip with burr-free PMMA microchannel fabricated by revolving tip-based micro-cutting. J. Mater. Process. Technol..

[16] Rodriguez W.G., Lara-Rodríguez Y.P., Ramos E.C.L., Ruiz A.S. Optimizing PMMA Surface: Roughness Study by CNC Milling. Proceedings of the 2020 IISE Annual Conference.

[17] Reichenbach I.G., Bohley M., Sousa F.J.P., Aurich J.C. (2019). Tool life criteria and wear behavior of single-edge ultra-small micro end mills. Precis. Eng..

[18] Jiao F., Cheng K. (2014). An experimental investigation on micromilling of polymethyl methacrylate components with nanometric surface roughness. Proc. Inst. Mech. Eng. Part B J. Eng. Manuf..

[19] Rahim M.S., Ehsan A.A. (2020). Optimization of Micromilling Parameters using Taguchi Method for the Fabrication of PMMA Based Microchannels. Int. J. Nanoelectron. Mater..

[20] Meng F., Cheng X., Sun Q., Xu R., Yang X. Study on Micromilling Processes for Polymethyl Methacrylate. Proceedings of the 6th International Conference on Information Engineering for Mechanics and Materials (ICIMM 2016).

[21] Yan Y., Zhou P., Wang H., Mao Y. (2020). Thermal Effect on Poly(methyl methacrylate) (PMMA) Material Removal in the Micromilling Process. Polymers.

[22] Yan Y., Sun Y., Li B., Zhou P. (2021). An experimental study of PMMA precision cryogenic micro-milling. Biomater. Polym. Horiz..

[23] Sun F., Guoyu F., Huo D. (2024). Computational and Experimental Analysis of Surface Residual Stresses in Polymers via Micro-Milling. Polymers.

[24] Şimşek Ü., Miyamoto H., Yalçınkaya T. (2025). Explicit Crystal Plasticity Modeling of Texture Evolution in Nonlinear Twist Extrusion. Crystals.

[25] Demiral M., Mamedov A. (2022). 3D SPH-FEM modelling of micro milling of Ti-6Al-4V. J. Adv. Manuf. Eng..

[26] Abeni A., Cappellini C., Attanasio A. (2023). A Novel 2D Micromilling FEM simulation strategy to optimize the flow stress law of IN625. Procedia CIRP.

[27] Bhople N., Mastu S., Satpal S. (2021). Modelling and analysis of cutting forces while micro end milling of Ti-alloy using finite element method. Int. J. Simul. Multidiscip. Des. Optim..

[28] Çoğun F., Yıldırım E., Arıkan M.A.S. (2017). Investigation on replication of microfluidic channels by hot embossing. Mater. Manuf. Processes.

[29] Sucularlı F., Arikan M.A.S., Yıldırım E. (2020). Investigation of process-affected zone in ultrasonic embossing of microchannels on thermoplastic substrates. J. Manuf. Processes.

[30] Mizuno Y., Nakamura K. (2011). Brillouin Scattering in Polymer Optical Fibers: Fundamental Properties and Potential Use in Sensors. Polymers.

[31] Wu X., Li L., He N. (2017). Investigation on the burr formation mechanism in micro cutting. Precis. Eng..

[32] Balázs B.Z., Szalay T., Takács M. Investigation of micro milled surface characteristics. Proceedings of the International Conference on Innovative Technologies.

[33] Hajiahmadi S. (2019). Burr size investigation in micro milling of stainless steel 316 L. Int. J. Lightweight Mater. Manuf..

[34] Piquard R., D’Acunto A., Laheurte P., Dudzinski D. (2014). Microend milling of NiTi biomedical alloys, burr formation and phase transformation. Precis. Eng..

[35] Black S., Chiles V., Lissaman A., Martin S. (1996). Principles of Engineering Manufacture.

[36] Şimşek Ü., Çoğun C. (2025). An Investigation of the Performance of Equal Channel Angular Pressed Copper Electrodes in Electric Discharge Machining. Crystals.

[37] García-Collado A., Medina-Sanchez G., Kumar Gupta M., Dorado-Vicente R. (2020). Application of the Finite Element Method to the Incremental Forming of Polymer Sheets: The Thermomechanical Coupled Model and Experimental Validations. Polymers.

[38] Brady J.F. (1971). Yielding Behavior of Glassy Amorphous Polymers. J. Appl. Phys..

[39] Lin H., Jin T., Lv L., Ai Q. (2019). Indentation Size Effect in Pressure-Sensitive Polymer Based on A Criterion for Description of Yield and Shear Transformation-Mediated Plasticity. Polymers.

